# AHCYL1 Is a Novel Biomarker for Predicting Prognosis and Immunotherapy Response in Colorectal Cancer

**DOI:** 10.1155/2022/5054324

**Published:** 2022-05-07

**Authors:** Xubin Li, Mengqiao Zhang, Xue Yu, Mei Xue, Xiaowei Li, Chao Ma, Wei Jia, Qiang Gao, Chunbo Kang

**Affiliations:** ^1^Department of General Surgery, Beijing Rehabilitation Hospital of Capital Medical University, Beijing, China; ^2^Department of Gastroenterology, Beijing Rehabilitation Hospital of Capital Medical University, Beijing, China; ^3^Department of Breast Surgery, Peking University International Hospital, Beijing, China

## Abstract

**Background:**

Colorectal cancer (CRC) is the third most frequent cancer worldwide. The AHCYL1 gene is required for CNV and has a close association with the tumor immune microenvironment. However, the predictive value of the AHCYL1 gene in patients with CRC remains unknown.

**Methods:**

AHCYL1 gene with prognostic potential was comprehensively analyzed. Next, using LASSO Cox regression, we fully examined and integrated the AHCYL1 and AHCYL1-related genes from TCGA database. Meanwhile, TCGA database was used to study the connection between AHCYL1 and the tumor immune microenvironment and tumor mutation burden (TMB) in CRC. The influence of AHCYL1 in tumor growth and the recruiting ability of CD8+ T cells were verified, respectively, in vivo and in tissues. To ascertain the connection between AHCYL1 and AHCYL1-related genes and the prognosis of CRC, a prognostic model was created and validated.

**Result:**

We demonstrated that AHCYL1 has a differential expression and patients with AHCYL1 deletion get shorter survival in CRC. Additionally, the tissues without AHCYL1 have a weaker ability to recruit the natural killer (NK) cell, CD8+ T cells, and tumor-infiltrating lymphocytes (TILs) and response to immunotherapy. Additionally, knockdown of AHCYL1 promoted tumor growth in the CRC mouse model and recruited lower CD8+ T cells in CRC tissues. TCGA database was used to classify patients into low- and high-risk categories based on the expression of four genes. Meanwhile, we discovered an association between the low-risk group and a lower TMB and a higher response to immunotherapy. Finally, a predictive nomogram based on these genes was developed and verified, yielding a C-index of 0.74.

**Conclusion:**

For CRC patients, the prognostic model based on AHCYL1 and AHCYL1-related genes showed a high predictive performance in terms of prognosis and immunotherapy response.

## 1. Introduction

Worldwide, colorectal cancer (CRC) is the third most frequent malignancy, and it is now the second leading cause of cancer-related mortality [1]. The advanced stage CRC has a poorer survival prognosis than the early stage [2, 3]. Despite human prolonged survival of CRC patients by surgical operation, radiotherapy, and chemotherapy, 5-year relative survival continues to be less than 50% [4]. Meanwhile, genetic susceptibility or influence of environmental factors contributes significantly to the development of CRC [5, 6].

Currently, copy number variation (CNV) is associated with CRC risk and prognosis [7]. CNV, a type of genetic structural variation, is often characterized by an increase or reduction in the copy number of genomic regions ranging in size from 1 kb to 3 Mb [8]. Adhesion, recognition, and communication activities in cells are all affected by the amplifications or deletions of copy numbers in cancer genomes [9, 10]. CRC is related to significant copy number modification in microsatellite stable (MSS), CpG island methylator phenotype- (CIMP-) negative CRCs, including the cancer-related genes, adenosylhomocysteine hydrolase (AHCY), and the upregulation of AHCY gene demonstrating a good correlation [11].

The adenosylhomocysteine hydrolase-like protein 1 (AHCYL1) is a paralog of the adenosylhomocysteine hydrolase (AHCY) [12]. As a novel intracellular protein, AHCYL1 may interact with the inositol 1,4,5-trisphosphate receptor, resulting in the release of intracellular calcium [13], which is involved in critical cellular activities [14]. Additionally, AHCYL1 can also participate in the progression of tumors. In the patients with epithelial ovarian cancer, AHYCL1 overexpression was associated with a good prognosis for survival [15]. In the cholangiocarcinoma without KRAS/BRAF mutations, the transcription of AHCYL1-FGFR2 did not encode a functional protein of relevance to cancer [16]. However, as the cancer with the same potential for KRAS/BRAF mutations, the function of AHCYL1 continues to be unknown in CRC.

In this study, we analyzed TCGA database for information on CRC samples to comprehensively establish the predictive usefulness of the AHYCL1 gene and its association to immunotherapy response.

## 2. Method and Materials

### 2.1. Data Collection

TCGA database (https://portal.gdc.cancer) was used to collect RNA-seq profiles of 647 CRC cases and 51 normal tissue samples. And we also extracted demographic data (age, days to death, vital status, clinical stage, mutations, copy number variations, and so on) of these samples.

### 2.2. Survival Analysis and Construction of Prognostic Model

The Kaplan-Meier curves were plotted by the “survminer” R package between different groups. Additionally, we conducted a univariate Cox analysis of overall survival (OS) using the R package “survival” to identify genes having a predictive value.

A 7 : 3 ratio of patients from TCGA dataset was randomly assigned to the training and testing groups. The training data was used to include the prognostic genes into the LASSO Cox regression utilizing the “glmnet” R package. To avoid the model overfitting problem, the penalty regularization parameter *λ* was set using tenfold cross-validation. Each patient's risk score was computed as follows: risk score = *e*^sum (each gene's expression × corresponding coefficient)^. Univariate and multivariate Cox regression analyses were used to examine if the risk score was an independent predictive predictor of OS when compared to other clinical characteristics.

An independent predictive factor nomogram and accompanying calibration maps were created using the “rms” R program. Using the R package “timeROC,” we conducted a time-dependent receiver operating characteristic (ROC) curve analysis to determine the prediction potential of the prognostic model.

### 2.3. Functional Enrichment Analysis and Immunotherapy Response Predictions

Gene set variation analysis (GSVA) was used to analyze the functional enrichment of risk scores and important genes using the “GSVA” R tools. Meanwhile, using single-sample gene set enrichment analysis (ssGSEA), the “gsva” R package was utilized to compute the infiltrating score of 16 immune cells.

Tumor immune dysfunction and exclusion (TIDE) (http://tide.dfci.harvard.edu/) is a computational technique for modeling tumor immune evasion by integrating the expression profiles of T cell malfunction and exclusion. In CRC patients, we employed the TIDE algorithm to forecast the clinical response to immune checkpoint blockade (ICB).

### 2.4. Cell Culture and Transfection

HT-29 cells were acquired from American Type Culture Collection (ATCC) and grew in RPMI-1640 media enriched with 10% fetal bovine serum (FBS; ExCell Bio, Shanghai, China). Mycoplasma contamination was found in these cell lines. Cell transfection was carried out according to the manufacturer's instructions using the Exfect 2000 transfection reagent (Vazyme, NJ, China).

### 2.5. Immunohistochemical (IHC) Staining

The AHCYL1 protein was immunocytochemically localized in the chicken oviduct using an anti-human AHCYL1 monoclonal antibody (cat. ab56761; Abcam) diluted 1 : 500 (1 g/ml) and an anti-human CD8+ T cell monoclonal antibody (cat. ab237709; Abcam) diluted 0.25 g/ml. As previously mentioned, antigen retrieval was carried out utilizing the boiling citrate technique [17].

### 2.6. Lentivirus and Stable Cell Line Generation

HT-29 cells were transfected with an AHCYL1 shRNA plasmid (cat. MR208502L3V, Origene), psPAX2, and pCMV-VSV-G, and the supernatant containing lentivirus particles was collected 48 hours after transfection in order to study the effects of the transfection on cell viability. To generate a cell line stably expressing AHCYL1 shRNA, HT-29 cells were expanded to 50–80% confluence prior to infection with the lentivirus and then treated with 1–3 g/ml puromycin 24 h later. We selected stable clones and performed western blot analysis to determine the expression of AHCYL1.

### 2.7. Western Blot

For 30 minutes on ice, cells were lysed using RIPA buffer supplemented with protease and phosphatase inhibitors. Centrifuge the cell lysates for 15 minutes at 1.2 × 10^4^ rpm, 4°C. The concentrations of total protein were measured using the Pierce BCA protein assay kit. Equivalent quantities of protein were isolated and transferred to PVDF membranes using 10% SDS polyacrylamide gels. After blocking the membranes with 5% BSA dissolved in TBST for 1 hour, they were incubated with primary antibodies (cat: ab178693, Abcam) overnight at 4°C. After washing, the membranes were incubated for 1 hour at room temperature with peroxidase-conjugated secondary antibodies. Clarity™ Western ECL Substrate was used to visualize immunoreactive bands. ImageJ software was used to determine the gray levels for each band.

### 2.8. Colorectal Cancer Xenograft

Hangzhou Ziyuan Experimental Animal Technology Co., Ltd offered four- to six-week-old BALb/c nu/nu male mice (Hangzhou, China). The mice were maintained in an infection-free environment. Without using any selection criterion, mice were randomly separated into independent groups of five (*n* = 10). Subcutaneous injections of HT-29 cells (2 × 10^6^/mice) expressing control (ctrl) and AHCYL1 shRNA were made into the right flanks of BALb/c nu/nu mice. Tumor development was tracked, and tumor volumes were determined using the formula *V* = (*L* × *W*^2^)/2 (*L*: length; *W*: width) as published before. The investigator was not blinded throughout the experiment or while evaluating the results.

### 2.9. Statistical Analysis

All statistical analyses were conducted using the R programming language (Version 4.0.3). The Kaplan-Meier analysis was used to compare the OS of various groups, followed by the log-rank test. All *P* values were calculated with a two-tailed distribution. If not otherwise mentioned, *P* < 0.05 was deemed statistically significant.

## 3. Results

### 3.1. The Copy Number Variations of AHCYL1 Were Associated with Differences in AHCYL1 Expression and the Prognosis of CRC Patients

**Figure 1:**
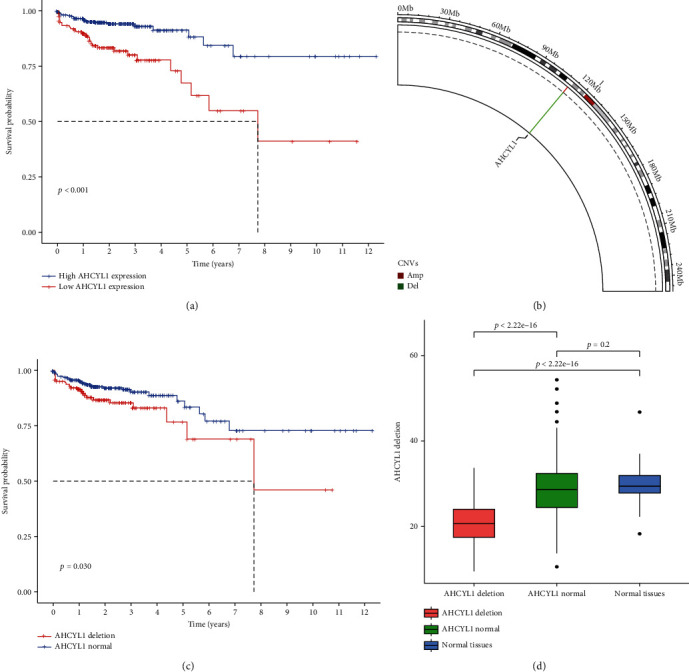
Expression variation and survival curves of AHCYL1 genes in colorectal cancer (CRC). (a) Kaplan-Meier graphs depicting the overall survival of patients with various AHCYL1 expression levels from TCGA database. (b) Using TCGA datasets, the position of AHCYL1 copy number variation (CNV) modification on chromosomes. (c) Kaplan-Meier curves comparing patients with AHCYL1 deletion to those with normal AHCYL1 expression from TCGA database. (d) AHCYL1 expression in various tissues.

Through the analysis from TCGA database, the Kaplan-Meier curve demonstrated that patients with high AHCYL1 expression had a significantly longer survival than those with low AHCYL1 expression in CRC (Figure 1(a)). We began by summarizing the occurrence of copy number variations (CNVs) and somatic mutations in the AHCYL1 gene in CRC patients. Around 29% of patients had a deletion of the AHCYL1 gene due to CNV, whereas the increase was less than 4% (Figure 1(b)). Since CNV is more common in CRC, we focused on the impact of copy number loss; the patients with AHCYL1 deletion have shorter OS than the normal patients (Figure 1(c)). To confirm the influence of the deletion of the AHCYL1 on gene expression, we analyzed TCGA database for different expressions. The results show that the deletion of AHCYL1 influenced straightly the expression of the AHCYL1 gene (Figure 1(d)).

### 3.2. Calculation of the Tumor Immune Microenvironment and Response to Cancer Immunotherapy

**Figure 2:**
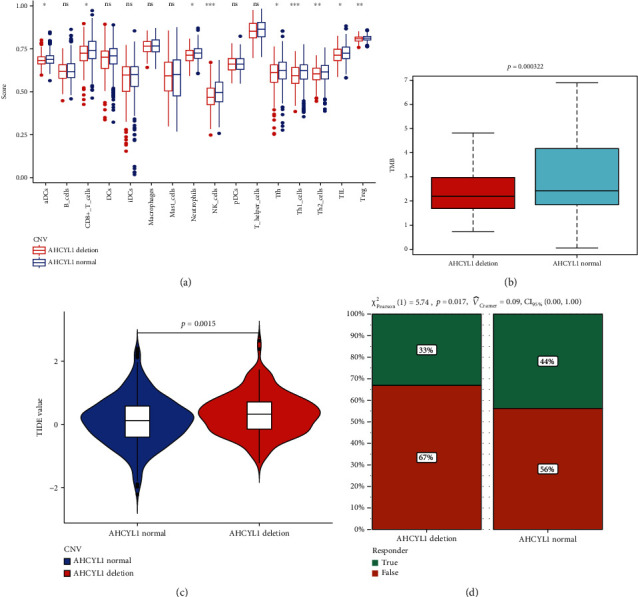
Relationship among AHCYL1, tumor mutation burden (TMB), and immunotherapy response to CRC patients. (a) The scores of 16 immune cells. (b) The TMB in tissues with different AHCYL1 expressions. (c) The value of the tumor immune dysfunction and exclusion (TIDE). (d) The response to immunotherapy (^∗^*P* < 0.05;  ^∗∗^*P* < 0.01;  ^∗∗∗^*P* < 0.001; ns: not significant).

To evaluate the relationship between tumor immune microenvironment and the deletion of AHCYL1, we analyzed the different evaluation indicators, like the recruitment of immune cell, TMB, TIDE, and the response for the immunotherapy. The result revealed that the group of AHCYL1 deletion has weaker ability to recruit the CD8+ T cell than the normal group (Figure 2(a)). And the TMB in the AHCYL1 deletion group is less than the normal (Figure 2(b)). For the estimation of immunotherapy, we also found that in the group of AHCYL1 deletion, patients had higher TIDE values, indicating higher potential of tumor immune evasion (Figure 2(c)), and were less likely to benefit from immunotherapy (Figure 2(d)).

### 3.3. AHCYL1 Knockdown Promoted Tumor Growth and Suppressed the Infiltration of CD8+ T Cells

**Figure 3:**
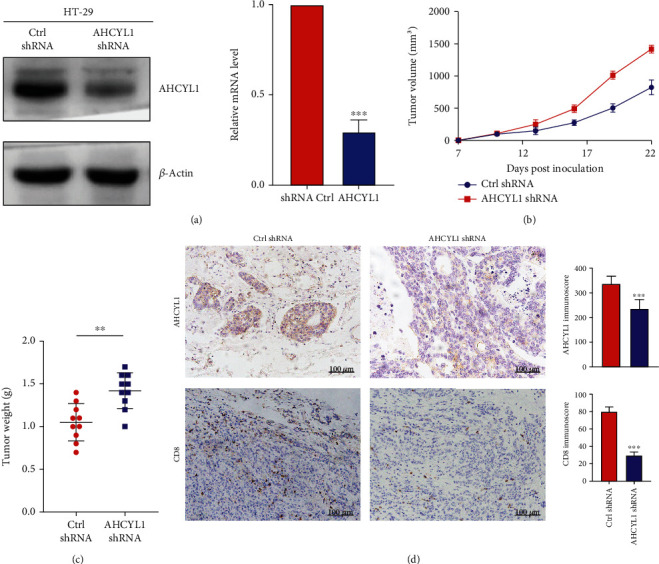
AHCYL1 knockdown inhibits tumor growth in CRC cancer xenografts. (a) Western blotting analysis of HT-29 cells expressing control (ctrl) or AHCYL1 shRNA. (b, c) HT-29 cells expressing ctrl or AHCYL1 shRNA have been implanted into BALB/c nu/nu mice; (b) tumor growth curve and (c) tumor weights have been measured. (d) Immunohistochemistry (IHC) analysis of CD8 positive T cells in HT-29 xenografts expressing ctrl or AHCYL1 shRNA. Data represents the mean ± SD, *n* = 10 per group. ^∗^*P* < 0.05,  ^∗∗^*P* < 0.01, and^∗∗∗^*P* < 0.001.

To further study the possible oncogenic function of AHCYL1 in CRC development in vivo, we generated human HT-29 CRC cells that were stably transfected with control (ctrl) or AHCYL1 shRNA (Figure 3(a)). To investigate the influence of AHCYL1 on tumor development, we implanted HT-29 cells into BALB/C nu/nu mice with or without AHCYL1 knockdown. In contrast to in vitro growth, we noticed that silencing AHCYL1 boosted tumor development (Figure 3(b)) and raised tumor weight (Figure 3(c)) much more than cells expressing ctrl shRNA. To verify the relationship between AHCYL1 and CD8+ T cells in CRC, we observed the infiltrating level of CD8+ T cells in the group with different expressions of AHCYL1 by IHC. We found that the group with low AHCYL1 expression gets weaker ability to recruit the CD8+ T cells (Figure 3(d)).

### 3.4. Constructing and Validating a Risk Model for CRC Based on AHCYL1-Associated Genes

**Figure 4:**
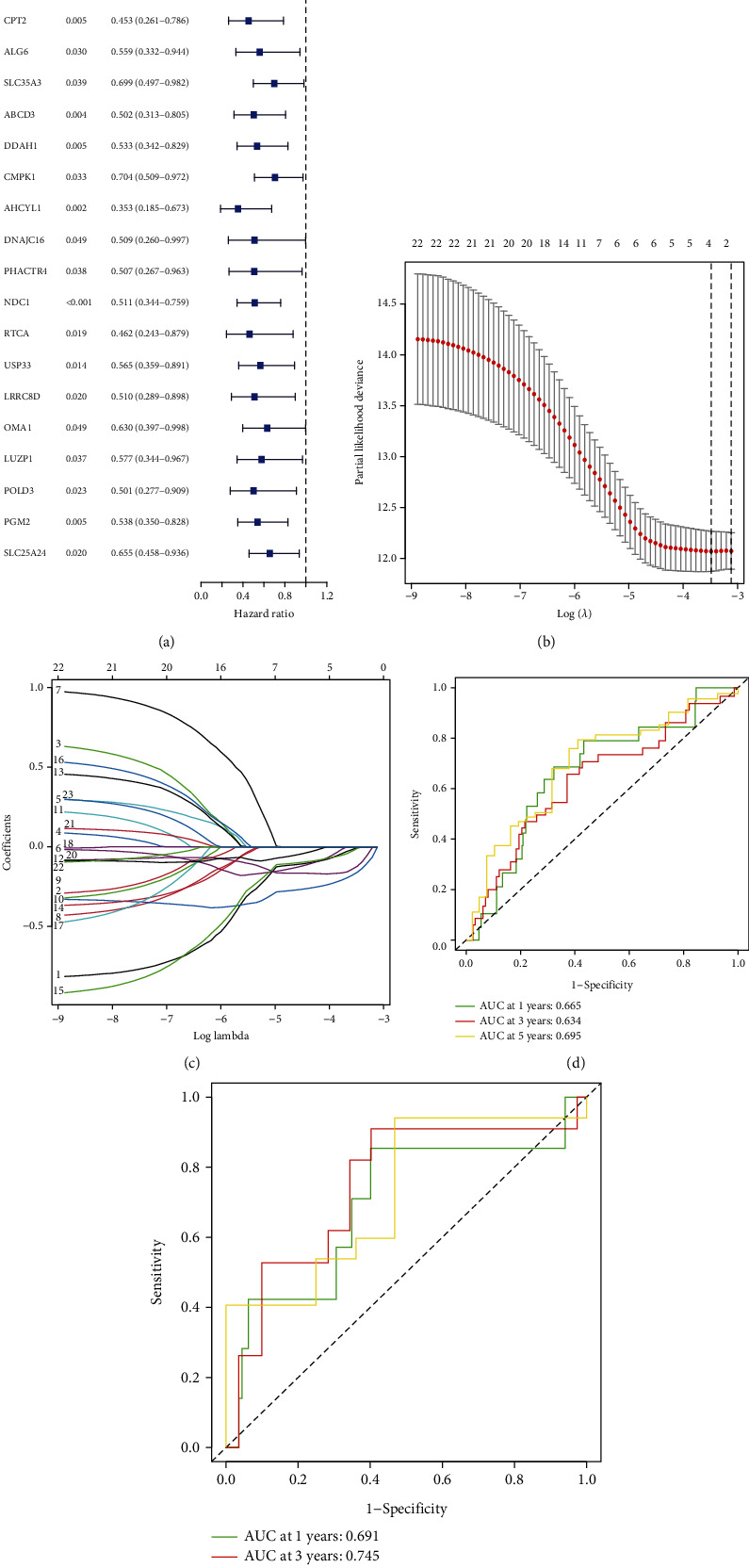
Risk model and the Kaplan-Meier curves for the OS in CRC patients based on RAPGEF2 and its related genes. (a) Univariate Cox regression analysis demonstrated a substantial correlation between the identified genes and clinical prognosis. (b) The profile of LASSO coefficients for 21 OS-related genes and the perpendicular imaginary line were drawn at the value determined by 4-fold cross-validation. (c) To cross-validate the error curve, the tuning parameters (log) of OS-related proteins were chosen. Perpendicular imaginary lines were drawn at the ideal value using the minimum and 1-se criteria. (d) Analysis of the receiver operating characteristic curve in the training group. (e) Analysis of the receiver operating characteristic curve in the testing group.

As we had shown the critical roles of the AHCYL1 deletion in the development of CRC, we desired to determine its prognostic value in CRC. We integrated prognosis and transcriptome data from CRC patients to identify genes that are closely related to the prognosis in CRC patients. We screened AHCYL1-associated genes from TCGA database through coexpression analysis, and coefficients greater than 0.50 were extracted for univariate Cox regression analysis to screen prognostic genes. As a result, 22 AHCYL1-related genes were significantly correlated to OS with *P* < 0.05 (Figure 4(a)). This comprehensive and effective risk signature for prognosis was created using LASSO Cox regression analysis on the 23 genes in the training set (Figures 4(b) and 4(c)). Four critical genes stucked out were NDC1, AHCYL1, DDAH1, and GNAI3. And risk score = *e*^((−0.005 × expression of GNAI3) + (−0.16 × expression of DDAH1) + (−0.123 × expression of AHCYL1) + (−0.01 × expression of NDC1))^. Additionally, the ROC analysis revealed that the risk model had a good predictive value for CRC patients in TCGA training and test sets (train set: 1-, 3-, and 5-year AUC = 0.665, 0.634, and 0.695; test set: 1-, 3-, and 5-year AUC = 0.691, 0.745, and 0.726; Figures 4(d) and 4(e)).

### 3.5. The Development and Validation of a Predictive Nomogram

**Figure 5:**
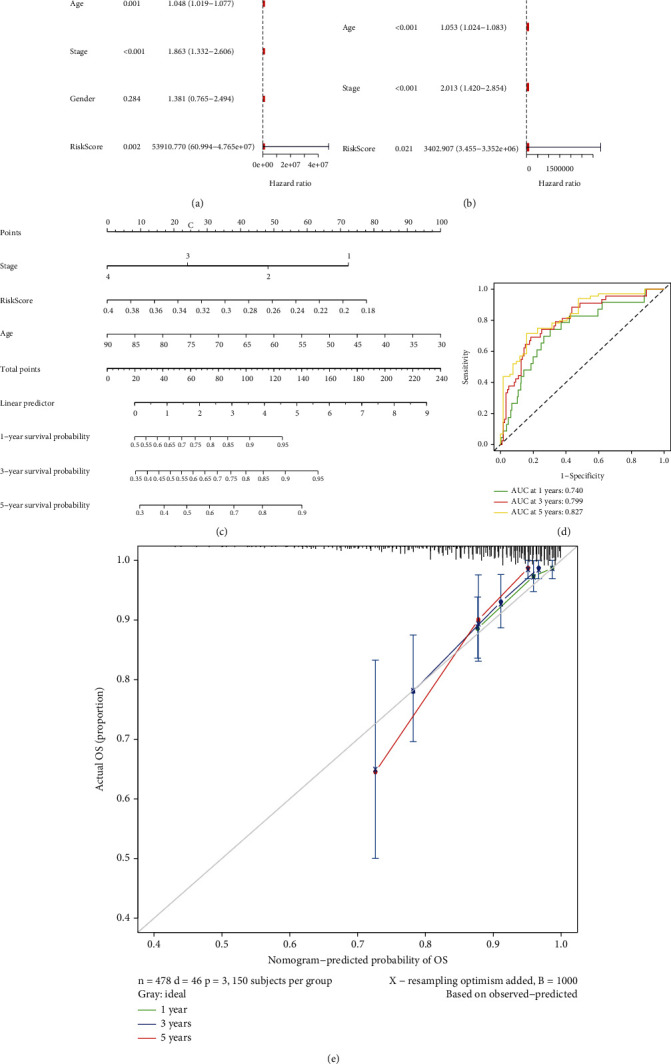
Construction and validation of a predictive nomogram. (a) The univariate Cox regression analysis' results. (b) Multivariate Cox regression analysis results. (c) A nomogram for predicting the overall survival (OS) of patients with CRC at 1, 3, and 5 years. (d) ROC analysis using TCGA database. (e) Nomogram calibration curves for OS prediction at 1, 3, and 5 years.

To assess the risk model's predictive power, we conducted univariate and multivariate Cox regression analyses and discovered that the risk score was a predictor of OS irrespective of other clinical characteristics (including gender, age, and TNM stage). Age (*P* = 0.001), TMN stage (*P* = 0.001), gender (*P* = 0.284), and risk score (*P* = 0.002) all had an effect on OS in univariate Cox proportional hazards regression analysis (Figure 5(a)). Multivariate Cox proportional hazards regression analysis revealed a strong association between age (HR = 1.053, *P* < 0.001), TNM stage (HR = 2.013, *P* = 0.028), and risk score (HR > 1000, *P* = 0.021) and overall survival in patients with CRC (Figure 5(b)). Meanwhile, a nomogram was devised to quantify the prediction of individual survival probability for 1, 3, and 5 years using these independent prognostic markers (Figure 5(c)). After that, the predictive value of the nomogram was evaluated using ROC curves. In TCGA database, the AUCs for 1-, 2-, and 3-year OS were 0.804, 0.807, and 0.805, respectively (Figure 5(d)). The nomogram's C-index was 0.74 (95% CI: 0.67-0.82). The calibration curves revealed a high degree of congruence between anticipated and observed OS at 1, 3, and 5 years (Figure 5(e)).

### 3.6. The Function of AHCYL1 Gene in the CRC Microenvironment

**Figure 6:**
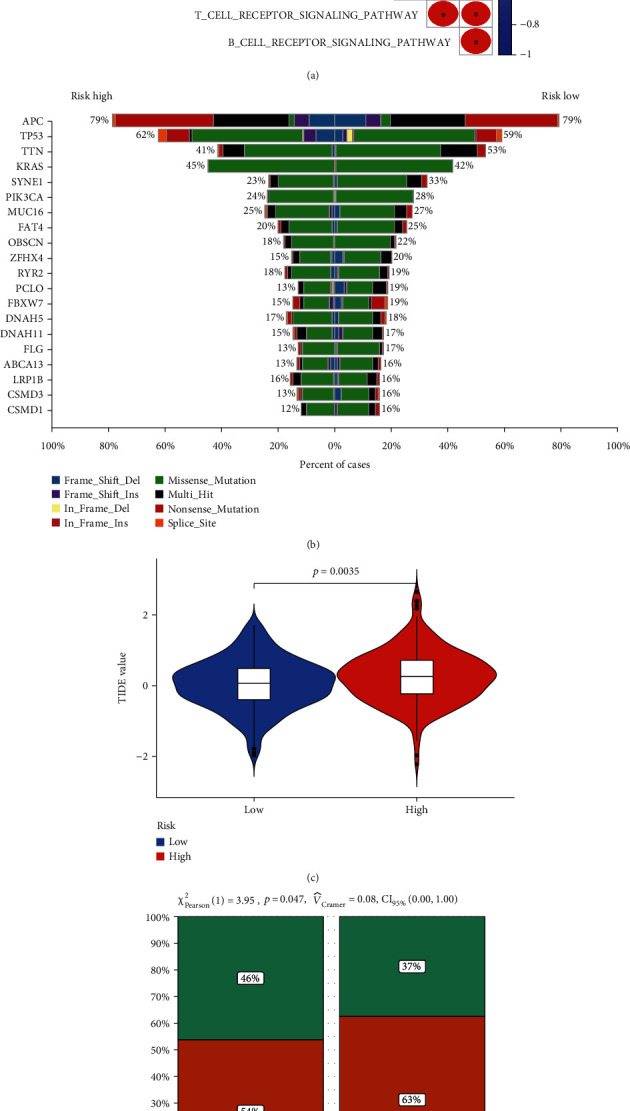
Relationship among risk model, TMB, TIDE, and immunotherapy response to CRC patients. (a) Correlation analysis to analyze the correlation between target gene AHCYL1 and its related prognostic genes in CRC. (b) The TMB in different risk groups. (c) The value of the TIDE from TCGA database. (d) The response to immunotherapy from TCGA database. (e) The value of the TIDE from ICGC database (^∗^*P* < 0.05;  ^∗∗^*P* < 0.01;  ^∗∗∗^*P* < 0.001).

After the prognostic analysis of the risk model, we need to find the profound function of the risk model. As Figure 6(a) shows, the AHCYL1 gene was associated with basic functions, like the TMB, B cell receptor signaling pathway, T cell receptor signaling pathway, P53 signaling pathway, DNA replication, risk score, and apoptosis. As seen in Figure 6(b), the low-risk score group had a greater load of tumor mutations than the high-risk score group (Figure 6(b)). Meanwhile, a similar outcome was seen in the TIDE value, with the group with a high-risk score having a greater TIDE value than the group with a low-risk score (Figure 6(c)). When it comes to immunotherapy response, the group with a high-risk score receives the false responder (Figure 6(d)).

## 4. Discussion

We used TCGA datasets to assess the expression of AHCYL1 genes and AHCYL1-associated genes in CRC samples and their correlation with the OS of CRC patients. Our result revealed that the expression of AHCYL1 was influenced straightly by the CNV. Meanwhile, because of the recruitment of CD8+ T cells and the low TMB, the patients with AHCYL1 gene deletion were insensitive to the immunotherapy leading to shorter survival. To accurately evaluate the influence of AHCYL1 gene in the tumor microenvironments, we screened out three AHCYL1-associated genes (GNAI3, DDAH1, and NDC1) and established a risk score model. The results of the relationship between the risk score model and tumor immune were similar to the AHCYL1 gene. It is the first prognostic model for CRC patients with the deletion of AHCYL1 genes.

At present, the CNV in 11q11 loss is identified as a potential candidate susceptibility variant for CRC [18]. A previous study showed KRAS mutation and CNV were concomitantly observed in partial CRC [19]. As important therapeutic targets, about 40% of CRC patients exhibit KRAS mutations, and the mutations are maintained throughout CRC progression [20]. Although KRAS mutations play crucial roles in CRC development and progression, the underlying mechanisms, especially concerning transcriptome variation, are still unclear. There is a research suggesting that high expression of NDC1 gene is enriched in the KRAS-related pathways in CRC [21]. In the cholangiocarcinoma without KRAS mutations, the transcription of AHCYL1 was not abnormal [16]. Similarly, in our observation for the tumor mutation burden, we clearly found the mutation rate of the KRAS gene gets higher in the group with high score risk. That indicated that the AHCYL1 and the AHCYL1-associated genes may become the potential therapeutic targets, but the mechanism among them needs to be further investigated.

Furthermore, the prognosis of CRC is related to immune cells such as CD8+ T cells and B cells [22, 23]. Tumor-infiltrating lymphocytes can improve the effect of immunotherapy [24]. Our result showed the GNAI3 gene was associated with the B cell reporter signaling pathway. A previous study discovered that the absence of GNAI3 in B cells could reduce chemoattractant receptor signaling [25]. Meanwhile, we also found the DDAH1 gene has a relationship with the T cell receptor signaling pathway. In the tumor immune microenvironment, DDAH1 overexpression inhibited the Wnt/GSK-3*β* signaling [26], which may enhance the recruitment of T cells [27]. That may influence the response of immunotherapy, which matches our result in the aspect of immunotherapy. Nonetheless, the function of ACHYL1 and its associated gene in tumor immune microenvironment remains further exploration.

## 5. Conclusion

In summary, we developed a new prognostic model based on ACHYL1 and its related genes in CRC, which showed significant predictive value for immunotherapy response in CRC patients. A future study is required to determine the mechanisms by which these genes interact with the tumor immune microenvironment in CRC.

## Data Availability

Data and materials used in this investigation may be obtained by contacting the corresponding author.
